# *MhNRAMP1* From *Malus hupehensis* Exacerbates Cell Death by Accelerating Cd Uptake in Tobacco and Apple Calli

**DOI:** 10.3389/fpls.2020.00957

**Published:** 2020-07-07

**Authors:** Weiwei Zhang, Songqing Yue, Jianfei Song, Mi Xun, Mengyuan Han, Hongqiang Yang

**Affiliations:** College of Horticulture Science and Engineering, Shandong Agricultural University, State Key Laboratory of Crop Biology, Tai’an, China

**Keywords:** cadmium, uptake, *Malus hupehensis*, natural resistance-associated macrophage protein, cell death, vacuolar processing enzyme

## Abstract

Excessive cadmium (Cd) damages plants by causing cell death. The present study discusses the function of natural resistance-associated macrophage protein (NRAMP) on cell death caused by Cd in *Malus hupehensis*. *MhNRAMP1* was isolated from *M. hupehensis* roots, and its protein was located in the cell membrane as a transmembrane protein characterized by hydrophobicity. *MhNRAMP1* expression in the roots was induced by Cd stress and calcium (Ca) deficiency. *MhNRAMP1* overexpression increased Cd concentration in yeasts and enhanced their sensitivity to Cd. Phenotypic comparisons of plants under Cd stress revealed that the growth of transgenic tobacco and apple calli overexpressing *MhNRAMP1* was worse than that of the wild type (WT). The Cd^2+^ influx of transgenic tobacco roots and apple calli was higher, and the recovery time of the Cd^2+^ influx to a stable state in transgenic apple calli was longer than that of the WT. Cd accumulation and the percentage of apoptotic cells in transgenic lines were higher. Correspondingly, the caspase-1-like and vacuolar processing enzyme (VPE) activities and *MdVPEγ* expression were higher in transgenic apple calli, but the expression levels of genes that inhibit cell death were lower than those in the WT under Cd stress. Moreover, the Cd translocation from the roots to leaves was increased after *MhNRAMP1* overexpression, but the Cd translocation from the leaves to seeds was not affected. These results suggest that *MhNRMAP1* exacerbated Cd-induced cell death, which was accomplished by mediating Cd^2+^ uptake and accumulation, as well as stimulating VPE.

## Introduction

Cadmium (Cd) is a toxic heavy metal and widespread pollutant found in natural environments. When exposed to an environment containing Cd, the Cd concentration in plants and animals easily exceeds the normal range due to the enrichment of Cd, which seriously threatens human health though the food chain (He et al., 2011). Cd in soil is easily taken up by plant roots and causes severe phytotoxicity to plants (Zare et al., 2018). Excessive Cd restricts nutrient uptake (Yamaguchi et al., 2016; Wang et al., 2017; Cengiz et al., 2019; Wu et al., 2019), decreases photosynthetic efficiency (Pereira de Araújo et al., 2017), delays seed germination (Zhang et al., 2014), and impairs plant growth and development (Bruno et al., 2017; Sofo et al., 2017), resulting in serious production reductions (Abbas et al., 2018; Guo et al., 2018; Chen et al., 2019). The current study shows that some orchard soils and even fruits have been contaminated by Cd, which threatens the growth of fruit trees and the edible safety of fruits (Cheng et al., 2015).

The entry of Cd into plant cells often accompanies the uptake of some essential micronutrients, such as iron (Fe), zinc (Zn), and manganese (Mn), because plant cells do not have a specific channel that binds to Cd only (Himeno et al., 2002; Verbruggen et al., 2009; Cai et al., 2019; Zou et al., 2020). The natural resistance-associated macrophage protein (NRAMP) family is one type of transporter responsible for metal uptake and translocation, such as Cd, Zn, Fe, and Mn (Cai et al., 2019; Wang et al., 2019c; Zou et al., 2020). *OsNRAMP6* in rice functions as a Fe and Mn transporter (Peris-Peris et al., 2017). *TpNRAMP3* in polish wheat transports Cd and Mn, but not Fe or Zn (Peng et al., 2018). *AhNRAMP1* is responsible for Mn and Zn uptake in peanut (Wang et al., 2019c). Due to the response to metal uptake and transport, NRAMPs regulate many environmental stress responses. NRAMP2 is required for *Arabidopsis* root growth under Mn deficiency (Gao et al., 2018). Knockout of *OsNRAMP5* produces low Cd-accumulating indica rice without compromising yield (Tang et al., 2017). *OsNRAMP1* regulates rice aluminum (Al) tolerance by controlling Al uptake (Li et al., 2014). *OsNRAMP6* contributes to disease resistance in rice by transporting Fe and Mn (Peris-Peris et al., 2017). *AtNRAMP6* possesses the ability to transport Cd and maintain intracellular Fe homeostasis in *Arabidopsis* under Fe-deficient conditions (Cailliatte et al., 2009; Li et al., 2019). NRAMPs have been well studied in rice because of their good control of Cd accumulation in grain; however, NRAMP in fruit trees is rarely reported. Previously, two NRAMPs were cloned from *Malus*. *MbNRAMP1* in *M. baccata* is responsible for Cd accumulation (Xiao et al., 2008). MxNRAMP1 in *M. xiaojingensis* promotes Fe absorption and increases plant resistance to Fe deficiency (Pan et al., 2015). Although *MbNRAMP1* increased yeast’s sensitivity to Cd, the mechanism has not been elucidated. The impact on fruit safety has not been reported.

Cell death is a stress response to Cd in plants. The excessive accumulation of reactive oxygen species (ROS) caused by Cd exacerbates DNA damage and increases cell membrane permeability and the loss of transmembrane potential (Li et al., 2003; Michelle et al., 2014; Li et al., 2017; Huybrechts et al., 2019; Wang et al., 2019b). Vacuolar rupture is a unique and important factor causing cell death in higher plants, which is mediated by vacuolar processing enzyme (VPE) (Yamada et al., 2020). VPE causes vacuolar rupture and initiates the proteolytic cascade leading to cell death and also possesses peptide ligation activity for producing cyclic peptides, which support developmental and environmental responses in plants (Jiang et al., 2019; Yamada et al., 2020). VPE is therefore recognized as a moderator in response to environmental tolerances. Expression of IbVPE1 from sweet potato in *Arabidopsis* affects leaf development, flowering time and chlorophyll catabolism (Jiang et al., 2019). Aluminum-induced cell death requires upregulation of NtVPE1 in tobacco (Kariya et al., 2018). The suppression of *OsVPE3* enhances salt tolerance by attenuating vacuole rupture during programmed cell death in rice (Lu et al., 2016). Our previous study showed that the expression of *MhVPEγ* exacerbated cell death in *Arabidopsis* under high temperature stress (Su et al., 2015). Correspondingly, cell death suppressors are also needed in plant responses to cell death, such as BAX inhibitor-1 (BI-1), Bcl-2-associated athanogene (BAG), and a defender against apoptosis death (DAD). BI-1 is a BCL-2- and BCL-XL-interacting anti-apoptotic protein capable of inhibiting BAX-mediated cell death induced by ROS in plants (Ishikawa et al., 2013; Ramiro et al., 2016). Plant BAG proteins function in cytoprotection under stress conditions (Kabbage and Dickman, 2008). DAD regulates light-induced cell death antagonistically through jasmonate and salicylate levels (Beaugelin et al., 2019). The expression of MhBAG and MhBI-1 was associated with root cell death in Malus hupehensis (Fan et al., 2013). The molecular machinery underlying cell death in plants is complex, and cell death caused by Cd has rarely been reported.

*Malus. hupehensis* Rehd. var. pingyiensis Jiang is a unique plant germplasm resource found in China that exhibits apomictic characteristics (Yang et al., 2008). It has waterlogging tolerance and is often used as an apple root stocks. In this study, the full-length of *MhNRAMP1* was obtained from *M. hupehensis* roots. The effects of *MhNRAMP1* on Cd-induced cell death, and Cd^2+^ uptake and accumulation were investigated using *MhNRAMP1*-overexpressing transgenic tobacco and apple calli. Phenotypic comparisons were made between wild type (WT) and transgenic plants under Cd stress. Moreover, the Cd translocation from roots to leaves and from leaves to seeds, and the expression of genes related to cell death were analyzed to investigate the possible regulatory mechanisms of *MhNRAMP1* involved in Cd-induced cell death and Cd translocation to seeds.

## Materials and Methods

### Plant Materials and Growth Conditions

*M. hupehensis* var. *pingyiensis* Jiang, tobacco (*Nicotiana tabacum* NC89), and apple calli (“Orin” cultivar) were used in this study. The germinated *M. hupehensis* seeds after stratification were cultivated in plastic pots filled with soil and grown in incubators under a 14/10 h light/dark photoperiod at 25/22°C. Seedlings with eight leaves were used in the experiment.

WT tobacco seedlings were grown in soil for 40 d, and the leaves were selected for genetic transformation. Both the WT and transgenic tobacco seedlings were grown in Murashige and Skoog’s (MS) agar medium and cultured in incubators under a 16 h light and 8 h dark photoperiod at 25°C.

The WT apple calli were used for genetic transformation. Both the WT and transgenic apple calli were grown on MS agar plates containing 0.5 mg L^-1^ IAA and 1.5 mg L^-1^ 6-BA at 24°C and were subcultured in 16 d intervals.

### MhNRAMP1 Isolation and Sequence Analysis

The complete mRNA sequence of *MhNRAMP1* was obtained by searching for *MdNRAMP1* (NCBI accession No.: XP_008354189.2) in the NCBI database and was amplified using MhNRAMP1-F and MhNRAMP1-R primers provided in **Table S1**, and then registered as MhNRAMP1 in GenBank (accession No.: MT035802.1). Complementary DNA (cDNA) was produced by reverse-transcription using the total RNA from *M. hupehensis* roots (Xu et al., 2012; Zhang et al., 2019a). PCR amplification was performed as follows: 5 min at 94°C, 35 cycles at 94°C for 30 s, 55°C for 30 s, 72°C for 2 min, and a final extension at 72°C for 10 min. The products were checked by sequencing (BGI, Shenzhen, China).

Multiple sequence alignments were performed using DNAMAN software (Lynnon Biosoft, USA). The full-length amino acid sequences of NRAMPs (**Figure S1**) were used for phylogenetic analysis. All of the acquired sequences were first aligned by ClustalX (version 1.83) software with the default parameters (Thompson et al., 1997). A neighbor-joining phylogenetic tree was constructed using MEGA6.0 software after 1,000 bootstraps (Tamura et al., 2007). ExPASy software was used to compute the isoelectric points (pI) and molecular weights (MW) (https://web.expasy.org/compute_pi/), as well as predict hydrophobicity (https://web.expasy.org/protscale/)and possible transmembrane helices (http://www.ch.embnet.org/software/TMPRED_form.html).

### Subcellular Localization of MhNRAMP1

The open reading frame sequence of *MhNRAMP1MhNRAMP1* was obtained using the NR1-F and NR1-R primers (**Table S1**) and was then cloned into the pROKII-GFP vector. Fluid from *Agrobacterium* containing the MhNRAMP1-GFP fusion vector or pROKII-GFP control vector was infiltrated into the WT tobacco leaves (Sheludko et al., 2007). The infiltrated tobacco was cultured in the dark for 2 d, and then the infected leaves were cut and observed under a Fluo-View FV1000confocal laser scanning microscope (Olympus, Japan).

### Experimental Treatments

The cultured *M. hupehensis* seedlings were irrigated with 1/2 Hoagland’s nutrient solution containing 50 μM CdSO_4_ for 14 d. The control *M. hupehensis* seedlings were irrigated with 1/2 Hoagland’s nutrient solution alone. Roots, stems, and leaves were collected after 0.5 h, 6 h, 12 h, 24 h, 48 h, 7 d, and 14 d. For Mn, Zn, Fe, and Ca stress responses, *M. hupehensis* seedlings were irrigated with 1/2 Hoagland’s nutrient solution containing 8 mM MgCl_2_, 8 mM ZnSO_4_, 8 mM FeCl_3_, or 8 mM CaNO_3_ for 24 h. For Mn-, Zn-, Fe-, and Ca-deficiency responses, *M. hupehensis* seedlings were irrigated with 1/2 Hoagland’s nutrient solution containing 0 mM Mn, 0 mM Zn, 0 mM Fe, or 0 mM Ca for 14?A3B2 show [#,32] ?> d. Lateral roots were subsequently sampled. Afterward, *MhNRAMP1* expression was measured.

### MhNRAMP1 Expression in Yeast

The *MhNRAMP1* ORF was amplified using NRAMP1-YF and NRAMP1-YR and then subcloned into the pYES2 empty vector. The *MhNRAMP1*-pYES2 recombinant vector and pYES2 empty vector were transformed into the BY4741 WT yeast strain. The transformed yeasts were selected on SD solid medium containing ampicillin. The transformed yeasts after sequence confirmation were cultured in liquid SD medium at 30°C until OD_600_ = 0.8. Each cell suspension was diluted to five sequential dilutions: 1, 0.1, 0.01, 0.001, and 0.0001. Five mL of each diluted cell suspension was spotted on plates with SD medium containing CdSO_4_ (0 or 10 μM) with 2% galactose. All plates were incubated at 30°C for 3 d to observe growth.

To further confirm metal sensitivity, 5 μL of yeasts (OD_600_ = 0.8) were cultured in 50 mL of SD liquid medium with 2% galactose and 5 μm CdSO_4_. OD_600_ values were measured every 6 h using a microplate spectrophotometer (Fisher Scientific, USA) with three replicates.

The Cd was extracted from the yeasts grown in SD liquid medium containing 5 μM CdSO_4_ for 84 h and was then determined by inductively coupled plasma-mass spectrometry (ICP-MS) (Fisher Scientific, USA).

### Tobacco Transformation and Treatment

The above PCR products were subcloned into the pROKII vector, and then the CaMV35S-MhNRAMP1 confusion vector was subsequently transformed into the LBA4404 *A. tumefaciens* strain. Then, the transgenic tobacco generation was obtained using the leaf disk transformation (Horsh et al., 1985). Three tobacco lines were selected for further analysis.

The sterilized tobacco seeds were sown on MS agar medium for 10 d, then transferred to plates containing 50 μm CdSO_4_, cultured for 14 d, and then photographed. Some of the seedlings were used for the Cd^2+^ flux analysis. The other seedlings were cultured until they were 80 d old and were then transferred to pots. The seedlings in pots were irrigated with 1/2 Hoagland’s nutrient solution (control) or 1/2 Hoagland’s solution containing 50 μM CdSO_4_ for 14 d. Then, the lateral roots, leaves, and seeds were collected to measure the Cd contents.

### Apple Calli Transformation and Treatment

The CaMV35S-MhNRAMP1 overexpression vector was used, and the *MhNRAMP1-*overexpression apple calli, NR1 and NR2, were generated through *Agrobacterium*-mediated genetic transformation (An et al., 2018).

The 8-day-old WT and transgenic apple calli (NR1, NR2) were transferred to MS agar medium containing CdSO_4_ and were photographed after 10 d. The fresh weight, Cd^2+^ flux, Cd contents, fluorescence, and gene expression levels were measured.

### Quantitative Real-Time PCR

The cDNA was obtained as described above in 2.2. The relative expressions of *MhNRAMP1*, *MdVPEγ* (NCBI accession No.: XM_008379705.2), *MdBI-1* (NCBI accession No.: XM_008358248.3), *MdBAG* (NCBI accession No.: XM_008378307.3), and *MdDAD* (NCBI accession No.: NM_001293917.1) were analyzed by quantitative real-time PCR (qRT-PCR) using SYBR Premix Ex Taq II (TaKaRa, Beijing, China). The expression levels of *MhNRAMP1* were normalized to the *Mh18S* gene, and the expression levels of *MdBI-*1, *MdBAG* *MdDAD*, and *MdVPEγ* were normalized to the *MdACTIN* gene. Primers are provided in **Table S1**.

### Net Cd^2+^ Flux Measured Using Non-Invasive Micro-Test Technology

The net Cd^2+^ flux of tobacco and apple calli was measured using non-invasive micro-test technology (NMT100 series) (YoungerUSA LLC, Amherst, MA, USA) [Xuyue (Beijing) Science & Technology Co., Ltd., Beijing, China]. The pre-pulled and silanized microsensor was made following the methods of Zhang et al. (2019a). The Cd^2+^ influx of tobacco roots was measured in fresh measuring solution for 25 min according to the methods described by Zhang et al. (2019a). The Cd^2+^ influx of apple calli was recorded for 25 min using the methods of Zhang et al. (2019b). imFluxes 2.0 software (YoungerUSA, LLC, Amherst, MA, USA) was used for data recording. Each treatment was repeated three times and all tests were repeated six times.

### Measurement of Cd Contents, TF, and Cd^2+^ Distribution

Dried tobacco samples and apple calli were digested with nitric acid in a MARS 6 microwave (CEM Microwave Technology, Ltd., Matthews, NC, USA). The Cd concentrations were determined using a NexION 300×, ICP-MS instrument (PerkinElmer, USA).

The translocation factor (TF) is defined as the transferability of Cd from one organ to another. TFs were calculated as the ratio of Cd concentration in the root, leaf or seed using the following equations:

$$\text{Cd}\;\text{transport}\;\text{from}\;\text{roots}\;\text{to}\;\text{leaves:}\;\text{TF}={\text{C}}_{\left[\text{leaf}\right]}/{\text{C}}_{\left[\text{root}\right]}\times 100\%,$$

$$\text{Cd}\;\text{transport}\;\text{from}\;\text{leaves}\;\text{to}\;\text{seeds:}\;\text{TF}={\text{C}}_{\left[\text{seed}\right]}/{\text{C}}_{\left[\text{leaf}\right]}\times 100\%,$$

where C_[leaf]_, C_[root]_, and C_[seed]_ are the Cd contents of the leaves, roots, and seeds, respectively.

The distribution of Cd^2+^ in apple calli cells was detected by a fluorescence probe Leadmium™ Green AM (Invitrogen, Carlsbad, CA, USA) according to Zhang et al. (2019b).

### Measurement of Apoptotic, Caspase 1-Like, and VPE Activities

Apoptotic activities were analyzed by determining the amount of annexin V positive cells for apoptosis or the amount of pI-positive cells for necrosis using a flow cytometer (PA, Partec, Germany) (Zhao et al., 2011). The apoptosis rate was expressed as the percentage of apoptotic cells (%).

Caspase 1-like activities were measured using a Caspase 1 Activity Assay kit and Micro- Bradford assay kit (Beyotime, Shanghai, China) following the manufacturer’s instructions. Total protein extracts (20 mg) were incubated consecutively for 2 h at 37°C with the synthetic tetrapeptide Ac-YVAD-pnitroaniline (pNA), during which, the addition of the substrate resulted in a signal caused by the caspase 1-dependent cleavage of the chromophore pNA from the labelled substrate. Caspase 1-like activities were measured at 405 nm. Enzymatic activities were expressed as the percentage of activity present in control extracts (%). Each measurement was conducted with three independent experiments.

VPE activities were measured following the methods with minor modifications described by Zhang et al. (2019a).

### Statistical Analysis

The data were analyzed by an analysis of variance (ANOVA) using SPSS v16.0 software (IBM, Chicago, IL, USA). All experiments were conducted in triplicate and the data are expressed as the mean ± standard deviation (SD). The confidence level for statistical significance was *p* ≤ 0.05. Figures were plotted using Origin v9.0 software (OriginLab, Northampton, MA, USA).

## Results

### Sequence Analysis and Expression Pattern Analysis of MhNRAMP1

The ORF of *MhNRAMP1* was 1,638 bp encoding a 545-amino-acid peptide. The deduced amino acid sequence was a 59.12 kDa polypeptide with a pI of 8.46. The MhNRAMP1 protein was predicted to be hydrophobic and had 11 strong transmembrane helices, implying that it was likely a transmembrane protein (**Figures S1A, B**).

Multiple alignments showed that the MhNRAMP1 protein was highly homologous to MdNRAMP1 and MdNRAMP6 from apple (99.08% identity), PbNRAMP6 from *Pyrus bretschneideri* (96.70%), PpNRAMP6 from *Prunus persica* (92.66%), and AtNRAMP1 from *Arabidopsis thaliana* (77.66%) (**Figure S1C**). The phylogenetic tree showed that MhNRAMP1 was homologous to a number of NRAMPs, but was most closely related to MdNRAMP1 and MdNRAMP6 (**Figure S1D**). The subcellular location indicated that MhNRAMP1 was localized in the cell membrane (**Figure S1E**).

**Figure 1:**
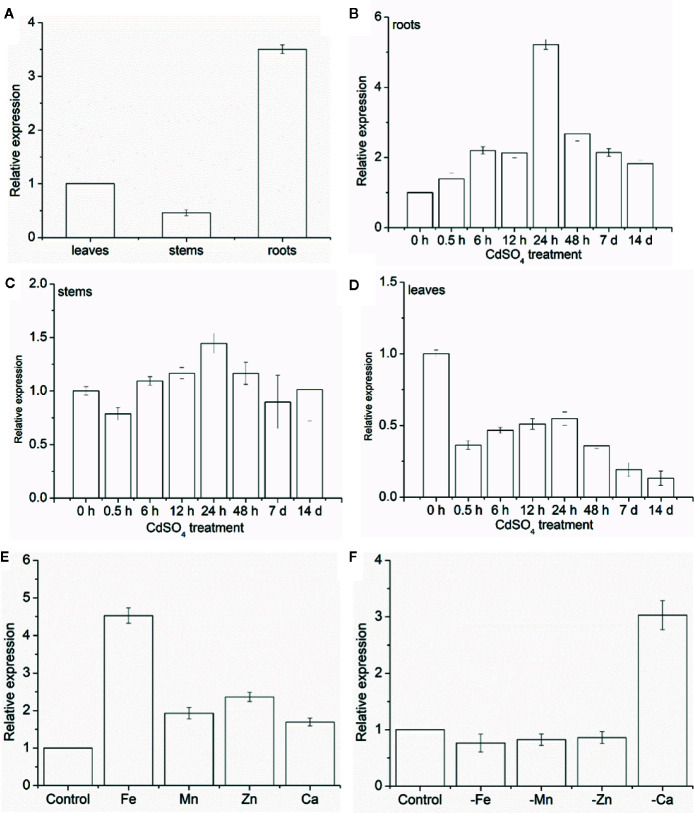
Expression pattern analysis of *MhNRAMP1*. **(A–D)** The relative expression of *MhNRAMP1* in *M. hupehensis* roots, stems, and leaves under normal **(A)** and 50 μm CdSO_4_ conditions **(B–D)**. **(E, F)** The relative expression of *MhNRAMP1* in *M. hupehensis* roots under over-supplementation **(E)** and deficiency **(F)** of nutrient metals, including Fe, Mn, Zn, and Ca. Data are presented as the mean ± standard derivation (SD) of three replicates; error bars represent the SD.

The expression levels of *MhNRAMP1* were examined in different plant tissues and treatments. Under normal conditions, *MhNRAMP1* was highly expressed in the roots (**Figure 1A**), but the expression varied greatly in different tissues as the Cd-treatment period progressed. *MhNRAMP1* in the roots was induced by Cd stress and was highly expressed after 24 h (**Figure 1B**). Despite starting at a low level, *MhNRAMP1* expression in the stems was slightly induced at 24 h under Cd stress (**Figure 1C**). However, *MhNRAMP1* in the leaves was inhibited under Cd stress (**Figure 1D**). These results indicated that *MhNRAMP1* was preferentially expressed in the roots, which were more sensitive to Cd than the stems or leaves.

The responses of *MhNRAMP1* in the roots to the over-supplementation or deficiency of nutrient metals, including Fe, Mn, Zn, and Ca, were also investigated. *MhNRAMP1* expression was significantly upregulated by Fe and was slightly upregulated by the supply of Mn, Zn, and Ca (**Figure 1E**). In contrast, *MhNRAMP1* expression was downregulated by Fe, Mn, and Zn deficiency, but was induced by Ca deficiency (**Figure 1F**).

### Overexpression of MhNRAMP1 Promoted Cd Uptake and Accumulation

**Figure 2:**
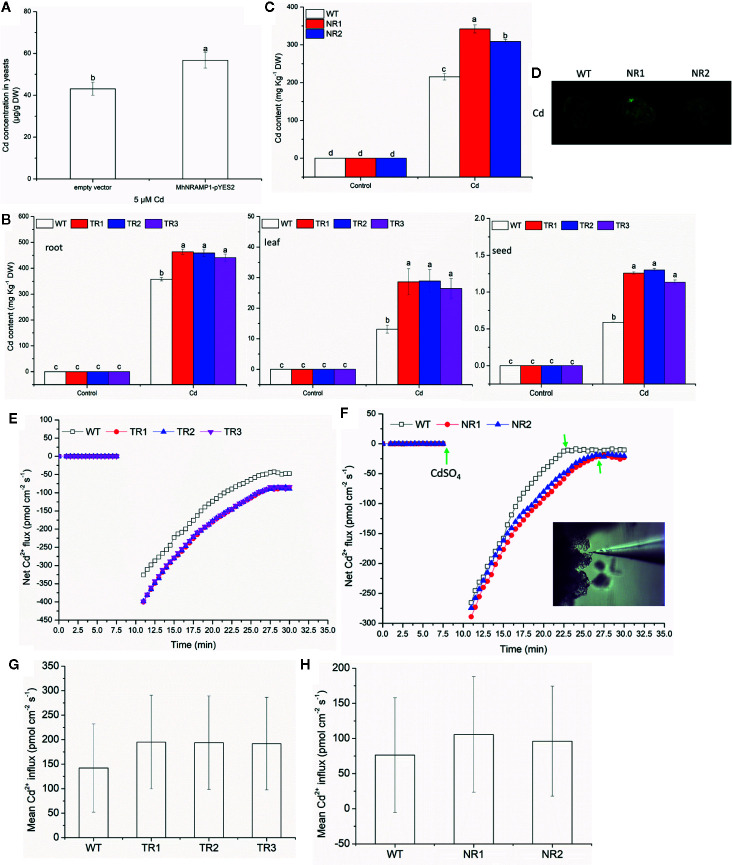
Effects of the overexpression of *MhNRAMP1* on Cd accumulation and Cd^2+^ uptake. **(A)** Cd concentrations in yeasts under CdSO_4_ treatment for 48 h. **(B)** the Cd contents in the root, leaf and seeds of WT and transgenic tobacco in the normal control and 50 μm CdSO_4_ treatment. **(C)** the Cd contents in the WT and transgenic apple calli under normal conditions and 50 μm CdSO_4_ treatment. **(D)** Cd distribution of the WT and transgenic apple calli in the 50 μm CdSO_4_ treatment. **(E)** the transient Cd^2+^ influx into the roots of WT and transgenic tobacco in the 50 μm CdSO_4_ treatment. **(F)** the transient Cd^2+^ influx into the WT and transgenic apple calli under normal conditions and the 50 μm CdSO_4_ treatment. **(G, H)** the mean Cd^2+^ influx in tobacco **(G)** and apple calli **(H)** in the 50 μm CdSO_4_ treatment. Data are presented as the mean ± SD of 3 independent measurements of three individual plants. Different letters above the columns indicate significant differences (*p* < 0.05).

To understand the response of *MhNRAMP1* to Cd, the transgenic yeasts, tobacco and apple calli were used to measure the Cd uptake and accumulation. **Figure 2A** illustrates that the transformed yeasts with *MhNRAMP1* had higher Cd concentrations than the yeasts with the empty vector. The Cd contents in the roots, leaves, and seeds of transgenic tobacco lines (TR1, TR2, TR3) that overexpressed *MhNRAMP1* were higher than those of the WT tobacco (**Figure 2B**). The transgenic apple calli (NR1 and NR2) that overexpressed *MhNRAMP1* also had higher Cd contents than the WT apple calli (**Figure 2C**). The Cd^2+^ distribution, as indicated by a fluorescent probe, showed that the cells of transgenic apple calli had stronger green fluorescent spots than the WT (**Figure 2D**).

The dynamic curves illustrating the net Cd^2+^ influx showed that the net Cd^2+^ influx into transgenic tobacco roots was higher than that of the WT tobacco (**Figure 2E**). The net Cd^2+^ influx into transgenic apple calli was similar to that of the WT before Cd addition (**Figure 2F**). After the addition of CdSO_4_, the net Cd^2+^ influx of transgenic apple calli immediately increased and was higher than that in the WT, and the net Cd^2+^ influx of transgenic apple calli reached a stable state after 27 min, which was later than the 23 min for the WT (**Figure 2F**). The mean Cd^2+^ influx of both transgenic tobacco and transgenic apple calli was also higher than that of their corresponding WTs (**Figures 2G, H**). Additionally, the Cd transport factor from roots to leaves (TF_leaf/root_) of the transgenic tobacco was higher compared to the WT; however, the TF from leaves to seeds (TF_seed/leaf_) of the transgenic tobacco was not different from the WT (**Table 1**). These results suggested that the overexpression of *MhNRAMP1* promoted Cd uptake and accumulation but did not affect Cd transport from the leaves to the seeds.

**Table 1:** The translocation factor of tobacco seedlings under CdSO_4_ stress.

Types	WT	TR1	TR2	TR3
TF_leaf/root_ (%)	3.67 ± 0.35^b^	5.08 ± 0.21^a^	5.17 ± 0.47^a^	4.89 ± 0.38^a^
TF_seed/leaf_ (%)	4.49 ± 0.35^a^	4.39 ± 0.47^a^	4.50 ± 0.02^a^	4.28 ± 0.38^a^

Data are presented as the mean ± standard derivation (SD) of three replicates; error bars represent the SD. Different of ^a^ and ^b^ indicates that the significance (p < 0.05).

### Overexpression of MhNRAMP1 Increased the Sensitivity of Yeasts, Tobacco, and Apple Calli to Cd

**Figure 3:**
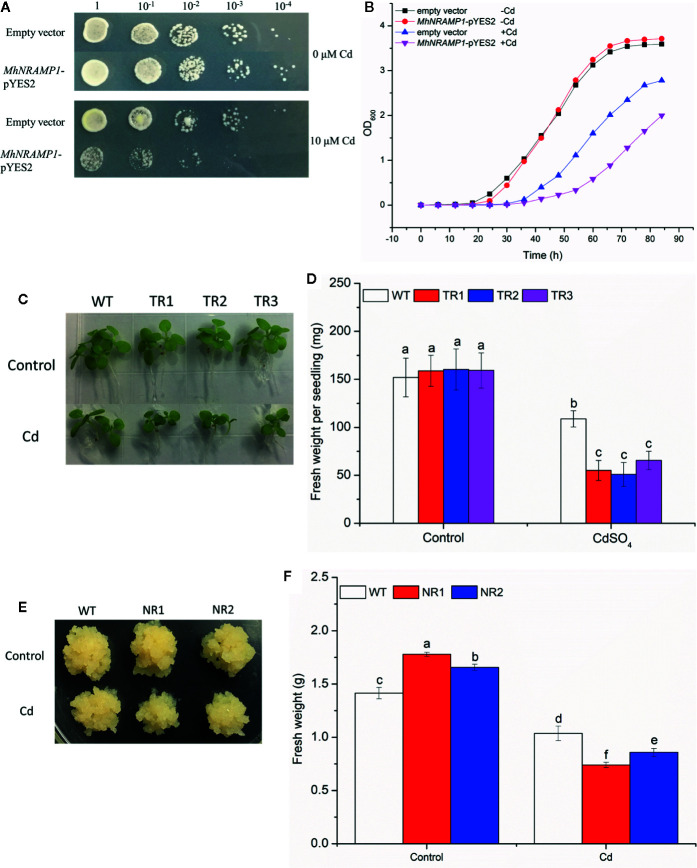
Effects of overexpression of *MhNRAMP1* on the growth of yeasts, tobacco, and apple calli. **(A)** the growth of yeasts on plates in SD medium with CdSO_4_. **(B)** the growth curves of yeasts in liquid SD medium with CdSO_4_. **(C)** phenotype comparisons between the WT and *MhNRAMP1-*overexpressing transgenic tobacco lines (TR1, TR2, and TR3) under normal conditions and CdSO_4_ stress. **(D)** the fresh weight of tobacco under normal conditions and 50 μm CdSO_4_ stress. **(E)** phenotype comparisons between the WT and *MhNRAMP1-*overexpressing transgenic apple calli (NR1 and NR2) and their fresh weight **(F)** under normal conditions and 50 μm CdSO_4_ stress. Data are presented as the mean ± SD of three independent measurements from three individual plants. Different letters above the columns indicate significant differences (*p* < 0.05).

The growth of yeasts, tobacco, and apple calli was measured to explore the role of *MhNRAMP1* in Cd sensitivity. No growth differences were observed between the transformed yeasts with *MhNRAMP1* and yeasts with the empty vector under 0 μm Cd stress (**Figure 3A**), but their growth was inhibited by 10 μm Cd, and the growth of transformed yeasts with *MhNRMAP6* was worse compared to the yeasts with the empty vector (**Figure 3A**). The growth of yeasts in liquid SD medium containing 5 μm Cd showed similar results (**Figure 3B**). Similarly, the transgenic tobacco seedlings under Cd stress displayed smaller sizes (**Figure 3C**) and lower fresh weight (**Figure 3D**). Notably, the NR1 and NR2 transgenic apple calli displayed slightly larger tissues than those of the WT under normal conditions; however, the transgenic apple calli were smaller than the WT under Cd stress (**Figure 3E**). The fresh weights of NR1 and NR2 under normal conditions were 1.26 and 1.17 times those of the WT, respectively. Under Cd stress, the fresh weights were 0.71 and 0.82 times those of the WT, respectively (**Figure 3F**). These results suggested that overexpression of *MhNRAMP1* enhanced plant sensitivity to Cd, was not conducive to growth under Cd stress.

### Overexpression of MhNRAMP1 Increased Cell Death Caused by Cd in Tobacco and Apple Calli

**Figure 4:**
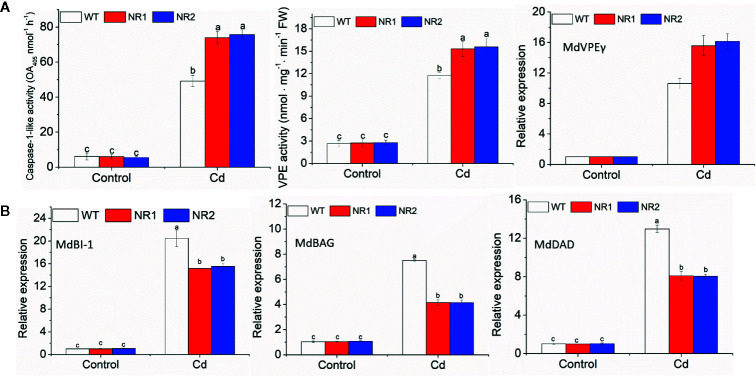
The caspase-1-like activities, VPE activities, and expression of *MdVPEγ* **(A)**, and the expression of *MdBI-1*, *MdBAG*, and *MdDAD* **(B)** in the WT and transgenic apple calli under Cd stress and normal conditions. Data are presented as the mean ± SD of three independent measurements from three individual plants. Different letters above the columns indicate significant differences (*p* < 0.05).

The transgenic apple calli and tobacco roots showed no significant difference in the percentage of apoptotic cells compared to their corresponding WTs under control conditions; however, the percentage of apoptotic cells under Cd stress in the transgenic apple calli and the transgenic tobacco roots was higher (**Table 2**). The gene expression and enzymes activities involved with cell death were measured in apple calli. Because MhVPEγ was found to have similar characteristics as caspase-1 in plants (Su et al., 2015), both the caspase-1-like activities and VPE activities of apple calli were detected and found to be higher in NR1 and NR2 transgenic apple calli under Cd stress, but they were not affected by the overexpression of *MhNRAMP1* under normal conditions (**Figure 4A**). The gene expression showed that the transgenic apple calli under Cd stress had higher *MdVPEγ* expression and lower expression levels of genes inhibiting cell death, including *MdBI-1*, *MdBAG*, and *MdDAD*, than the WT (**Figures 4A, B**). Furthermore, these gene expressions were not different between transgenic apple calli and the WT under normal conditions (**Figures 4A, B**).

**Table 2:** The percentages of apoptotic cells in apple calli and tobacco roots under normal conditions (control) and CdSO_4_ treatment.

	Treatment	Rate of cell death (%)				
**Apple calli**		**WT**	**NA1**	**NA2**		
	Control	3.44 ± 0.29^a^	3.33 ± 0.15^a^	3.49 ± 0.22^a^		
	Cd	14.93 ± 0.98^a^	19.03 ± 0.95^b^	20.16 ± 0.85^b^	
**Tobacco**		**WT**	**TR1**	**TR2**	**TR3**	
	Control	2.33 ± 0.21^a^	2.15 ± 0.42^a^	2.49 ± 0.19^a^	2.28 ± 0.27^a^	
	Cd	8.54 ± 0.58^a^	12.96 ± 0.47^b^	12.77 ± 0.36^b^	13.11 ± 0.42^b^	

Data are presented as the mean ± standard derivation (SD) of three replicates; error bars represent the SD. Different of ^a^ and ^b^ indicates that the significance (p < 0.05).

## Discussion

NRAMPs regulate plant stress responses and have been cloned from apple rootstocks, including *M. baccata* (Xiao et al., 2008) and *M. xiaojingensis* (Pan et al., 2015). In the present study, *MhNRAMP1* was isolated from *M. hupehensis* roots and was found to be highly homologous to *MdNRAMP1* and *MdNRAMP6* from apple (**Figure S1**). As a metal transporter, NRAMP genes were found to be induced by metals, including Fe (Peris-Peris et al., 2017), Mn (Wu et al., 2016), and Cd (Peng et al., 2018). *MhNRAMP1* was significantly induced by the oversupply of Fe and only slightly induced by the oversupply of Mn, Zn, or Ca (**Figure 1**), suggesting that *MhNRAMP1* might be more sensitive to Fe stress than to Mn, Zn, or Ca. When exposed to Ca deficiency, *MhNRAMP1* expression was upregulated, which contrasted its downregulation due to Fe, Mn, or Zn deficiency (**Figure 1**). Similarly, Picard et al. (2000) demonstrated that NRAMPs do not transport Ca^2+^, as the amino acid of NRAMPs has low effective selectivity on Ca (Bozzi et al., 2016). Thus, it was inferred that the significant increase of *MhNRAMP1* expression levels may be caused by its participation in Ca deficiency stress responses.

Cd is a preferred substrate of NRAMPs (Bozzi et al., 2016), which lead to long-term, low-dose Cd that could induce NRAMP responses. Previous studies have shown that NRAMP genes were induced by Cd (Ullah et al., 2018; Meena et al., 2018). In the present study, *MhNRAMP1* in the roots was induced by Cd, and its expression after 24 h was more sensitive to Cd (**Figure 1**). The effects of Cd on *MhNRAMP1* expression in the stems was not significant, but Cd inhibited the expression of *MhNRAMP1* in the leaves (**Figure 1**), implying that *MhNRAMP1* may play a more important role in the roots.

Many NRAMPs have Cd transport activity besides Fe and Mn. *MbNRAMP* of *M. baccata* was induced by Cd and was able to increase Cd accumulation and Cd sensitivity in yeast cells (Xiao et al., 2008). Previous studies reported that *NRAMP6* overexpression enhanced Cd accumulation in plants (Wang et al., 2019a; Chen et al., 2017). In the present study, *MhNRAMP1* overexpression increased Cd accumulation in yeasts (**Figure 2**) and also increased Cd accumulation in transgenic tobacco and apple calli. Correspondingly, the growth of transgenic yeasts was worse, and the transgenic tobacco and apple calli displayed smaller sizes and lower fresh weights under Cd stress (**Figures 2** and **3**). Therefore, the overexpression of *MhNRAMP1* increased Cd sensitivity in yeasts, tobacco, and apple calli by stimulating Cd accumulation. Importantly, the net Cd^2+^ influx in both tobacco roots and apple calli was increased by the overexpression of *MhNRAMP1* (**Figure 2**), which directly demonstrated that *MhNRAMP1* controlled Cd^2+^ uptake. Moreover, *MhNRAMP1* overexpression delayed the stabilization of the Cd influx to a relatively low level, which may explain the greater Cd^2+^ uptake observed in transgenic seedlings (**Figure 2**). These findings suggested that the sensitivities of transgenic yeasts, tobacco, and apple calli were likely due to the increase in Cd uptake regulated by *MhNRAMP1*.

Cell death is a typical physiological characteristic of Cd-damaged plants (Huybrechts et al., 2019). The percentage of apoptotic cells in tobacco roots and apple calli increased after Cd stress, and transgenic tobacco and apple calli overexpressing *MhNRAMP1* had higher percentages of cell death (**Table 2**). These higher percentages of cell death were certainly caused by higher Cd accumulation in transgenic lines. However, the contribution mediated by VPE should not be ignored. Both the VPE activities and capase-1-like activities exhibited higher levels in transgenic apple calli under Cd stress (**Figure 4**). The expression levels of *MdBI-1*, *MdDAD*, and *MdBAG*, which inhibit cell death, were down-regulated in transgenic apple calli (**Figure 4**). The regulation of enzyme activities and gene expression was probably caused by higher Cd accumulation in transgenic apple calli under Cd stress because the levels of enzyme activities and gene expressions in transgenic apple calli were similar to those in the WT under normal conditions (**Figure 4**). However, the effect of the stimulation of overexpression of MhNRAMP1 on enzymes activities and gene expression that regulate cell death cannot be excluded. *MhNRAMP1* promoted Cd-induced cell death mediated by VPE by accelerating Cd uptake.

The overexpression of *MhNRAMP1* in tobacco is due to the Cd transport from roots to leaves. Due to the increase of environmental Cd, the Cd contents of tobacco roots, seeds, and leaves increased in the present study (**Figure 2**). However, the Cd TF from leaves to seeds and from roots to leaves was different from that from leaves to seeds. The Cd TF from roots to leaves in transgenic tobacco was higher than the WT; however, the Cd TF from leaves to seeds in transgenic tobacco was similar to that of the WT (**Figure 2**). These results reflected the fact that *MhNRAMP1* played a role in Cd transport from the roots to leaves but did not influence the Cd transport from leaves to seeds, which agreed with the previous inference that *MhNRAMP1* tends to play a more important role in the roots. Therefore, we infer that *MhNRAMP1* might be similar to *OsNRAMP1* in assisting with Cd uptake and xylem loading for root to shoot mobilization, thus minimizing the Cd content in grain (Tiwari et al., 2014). Based on these effects, *MhNRAMP1* may be used in Cd enrichment without threatening the food safety of apple fruits.

## Conclusion

In the present study, *MhNRAMP1* was isolated from *M. hupehensis* roots and was mainly expressed in roots. *MhNRAMP1* overexpression increased Cd accumulation in yeasts and enhanced their sensitivity to Cd. Transgenic tobacco and apple calli overexpressing *MhNRAMP1* displayed greater Cd^2+^ uptake and Cd accumulation, and exhibited worse growth under Cd stress. Transgenic tobacco and apple calli also had higher cell death. The overexpression of *MhNRAMP1* in apple calli up-regulated VPE activities and *MdVPEγ* expression and inhibited the expression of genes involved in anti-cell death. These findings are the first to suggest that NRAMPs from *Malus* regulate Cd^2+^ uptake in plant roots, thereby controlling Cd accumulation in plants. This preliminary exploration into the molecular mechanisms underlying Cd-induced cell death in *M. hupehensis* is an important finding for future studies. However, the manner in which *MhNRAMP1* regulates VPE activities or its expression requires further investigation.

## Data Availability Statement

The datasets presented in this study can be found in online repositories. The names of the repository/repositories and accession number(s) can be found below: https://www.ncbi.nlm.nih.gov/nuccore/MT035802.1/, MT035802.1, https://www.ncbi.nlm.nih.gov/protein/XP_008354189.2, XP_008354189.2, https://www.ncbi.nlm.nih.gov/nuccore/XM_008379705.2, XM_008379705.2, https://www.ncbi.nlm.nih.gov/nuccore/XM_008358248.3, XM_008358248.3, https://www.ncbi.nlm.nih.gov/nuccore/XM_008378307.3, XM_008378307.3, https://www.ncbi.nlm.nih.gov/nuccore/NM_001293917.1, NM_001293917.1.

## Author Contributions

WZ designed the experiments and drafted the manuscript. SY, MX, and JS conducted the gene cloning and transformations, and performed the ion flux and ion content experiments. SY and MH analyzed the data. WZ and HY were the project leaders.

## Funding

This work was financially supported by the National Natural Science Foundation of China (grant No. 31801838), the National Key R&D Program of China (grant No. 2019YFD1000103), and the Shandong Provincial Natural Science Foundation of China (grant No. ZR2017BC024).

## Conflict of Interest

The authors declare that the research was conducted in the absence of any commercial or financial relationships that could be construed as a potential conflict of interest.

## Supplementary Material

Figure S1 The sequence analysis and subcellular location of MhNRAMP1. A–B, analysis of hydrophobicity **(A)** and transmembrane **(B)** of the MhNRAMP1 protein. **C**, multiple alignment of MhNRAMP1-deduced amino acid sequences and several NRAMP proteins from other plants, including apples (MdNRAMP6, XP_008354189.1), *Pyrus bretschneideri* (PbNRAMP6, XP_009354189.1), *Prunus persica* (PpNRAMP6, XP_007217247.1), *Prunus avium* (PaNRAMP6, XP_021821838.1), *Citrus sinensis* (CsNRAMP6, XP_006493353.1), *Fragaria vesca* subsp. *vesca* (FvsNRAMP6, XP_004303243.1), and *Arabidopsis thaliana* (AtNRAMP6, NP_173048.3). **D**, Phylogenetic tree construction of MhNRAMP1 in *Malus hupehensis* with NRAMPs from 18 other plant species, including apples (MdNRAMP2, XP_008351981.1; MdNRAMP3, XP_008361431.1; MdNRAMP5, XP_008361001.1; MdNRAMP6, XP_008354189.1), *Arabidopsis thaliana* (AtNRAMP1, NP_178198.1; AtNRAMP2, NP_175157.1; AtNRAMP3, NP_179896.1; AtNRAMP4, NP_201534.1; AtNRAMP5, OAO96785.1; AtNRAMP6, NP_173048.3), *Cajanus cajan* (CcNRAMP6, XP_020232276.1), *Citrus sinensis* (CsNRAMP6, XP_006493353.1), *Fragaria vesca* subsp. *vesca* (FvsNRAMP6, XP_004303243.1), *Glycine max* (GmNRAMP6, XP_003543701.1), *Glycine soja* (GsNRAMP6, KHN31883.1), *Juglans regia* (JrNRAMP6, XP_018846317.1), *Medicago truncatula* (MtNRAMP6, XP_013465598.1), *Prunus avium* (PaNRAMP6, XP_021821838.1), *Pyrus bretschneideri* (PbNRAMP6, XP_009354189.1), *Prunus mume* (PmNRAMP6, XP_008228792.1), *Prunus persica* (PpNRAMP6, XP_007217247.1), *Populus trichocarpa* (PtNRAMP6, XP_006368514.1), Rosa chinensis (RcNRAMP6, XP_024185381.1), and *Theobroma cacao* (TcNRAMP6, XP_017977974.1). **E**, subcellular location of MhNRAMP1.

Table S1 Primer sequences used for cloning, subcellular localization, vector construction, and expression analysis.
