# Supplementary figures and images for: MhNRAMP1 From Malus hupehensis Exacerbates Cell Death by Accelerating Cd Uptake in Tobacco and Apple Calli
Source: Front Plant Sci. 2020 Jul 7;11:957. doi: 10.3389/fpls.2020.00957 (PMC7358555; doi:10.3389/fpls.2020.00957)

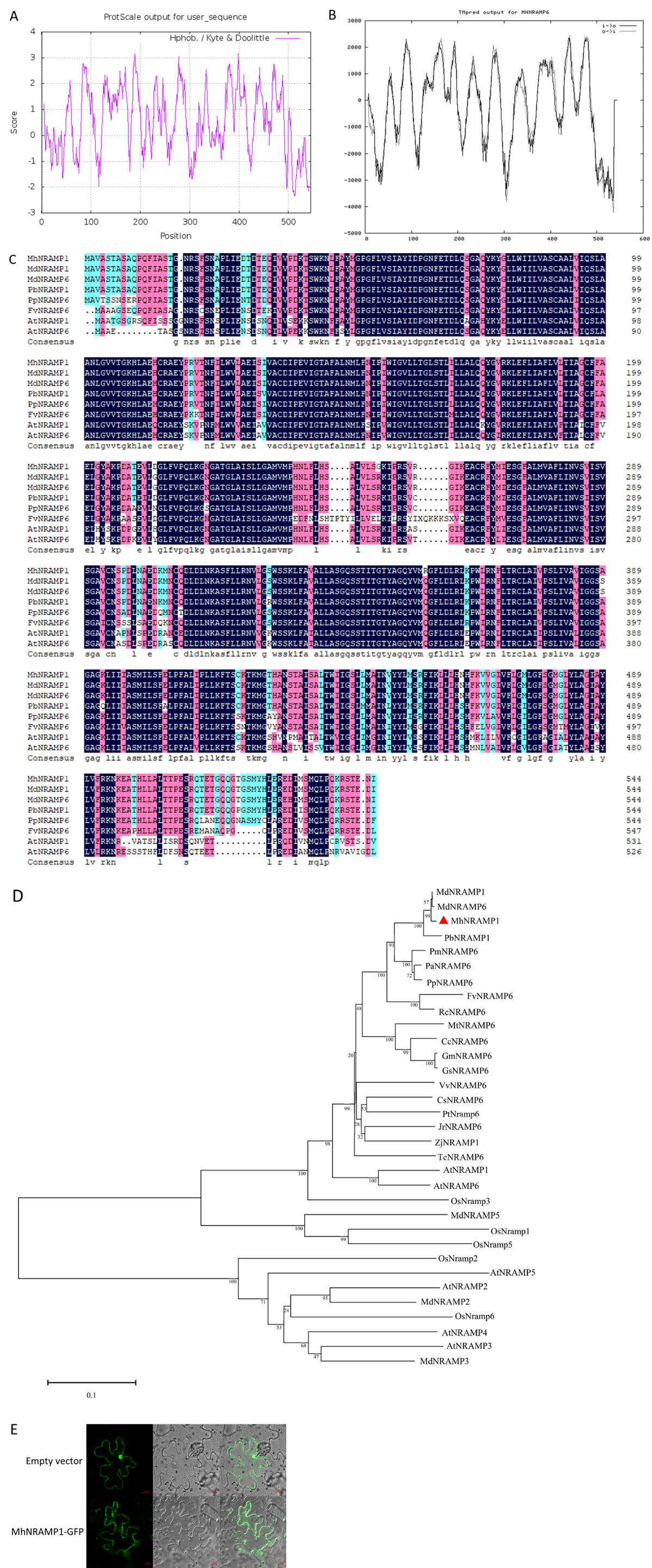

Supplement: Figure S1 — The sequence analysis and subcellular location of MhNRAMP1. A–B, analysis of hydrophobicity (A) and transmembrane (B) of the MhNRAMP1 protein. C, multiple alignment of MhNRAMP1-deduced amino acid sequences and several NRAMP proteins from other plants, including apples (MdNRAMP6, XP_008354189.1), Pyrus bretschneideri (PbNRAMP6, XP_009354189.1), Prunus persica (PpNRAMP6, XP_007217247.1), Prunus avium (PaNRAMP6, XP_021821838.1), Citrus sinensis (CsNRAMP6, XP_006493353.1), Fragaria vesca subsp. vesca (FvsNRAMP6, XP_004303243.1), and Arabidopsis thaliana (AtNRAMP6, NP_173048.3). D, Phylogenetic tree construction of MhNRAMP1 in Malus hupehensis with NRAMPs from 18 other plant species, including apples (MdNRAMP2, XP_008351981.1; MdNRAMP3, XP_008361431.1; MdNRAMP5, XP_008361001.1; MdNRAMP6, XP_008354189.1), Arabidopsis thaliana (AtNRAMP1, NP_178198.1; AtNRAMP2, NP_175157.1; AtNRAMP3, NP_179896.1; AtNRAMP4, NP_201534.1; AtNRAMP5, OAO96785.1; AtNRAMP6, NP_173048.3), Cajanus cajan (CcNRAMP6, XP_020232276.1), Citrus sinensis (CsNRAMP6, XP_006493353.1), Fragaria vesca subsp. vesca (FvsNRAMP6, XP_004303243.1), Glycine max (GmNRAMP6, XP_003543701.1), Glycine soja (GsNRAMP6, KHN31883.1), Juglans regia (JrNRAMP6, XP_018846317.1), Medicago truncatula (MtNRAMP6, XP_013465598.1), Prunus avium (PaNRAMP6, XP_021821838.1), Pyrus bretschneideri (PbNRAMP6, XP_009354189.1), Prunus mume (PmNRAMP6, XP_008228792.1), Prunus persica (PpNRAMP6, XP_007217247.1), Populus trichocarpa (PtNRAMP6, XP_006368514.1), Rosa chinensis (RcNRAMP6, XP_024185381.1), and Theobroma cacao (TcNRAMP6, XP_017977974.1). E, subcellular location of MhNRAMP1. [file Image_1.tif]
