# Supplementary material for: MhNRAMP1 From Malus hupehensis Exacerbates Cell Death by Accelerating Cd Uptake in Tobacco and Apple Calli
Source: Front Plant Sci. 2020 Jul 7;11:957. doi: 10.3389/fpls.2020.00957 (PMC7358555; doi:10.3389/fpls.2020.00957)
Supplement: Table S1 — Primer sequences used for cloning, subcellular localization, vector construction, and expression analysis. [file Table_1.docx]

Table 1 Primer sequences used for cloning, subcellular localization, vector construction, and expression analysis

| Genes | Primer sequence (5′- 3′) |
| --- | --- |
| *MhNRAMP1-*F | ATGGCTGTGGCCAGTACGG |
| *MhNRAMP1-*R | GTCGATATTTTCGGTAGACC |
| NR1-F | CGGGATCC ATGGCTGTGGCCAGTACGG BamHI |
| NR1-R | GGGGTACC GTCGATATTTTCGGTAGACC KpnI |
| *MhNRAMP1*-QF | CAGCTCTCACTTGGATCATT |
| *MhNRAMP1*-QR | TAGAGCCAAAAGATGCGTAG |
| *Mh18S*-F | AAACGGCTACCACATCCA |
| *Mh18S*-R | CACCAGAC TTGCCCTCCA |
| *NRAMP1-YF* | GCTCTAGA ATGGCTGTGGCCAGTACGG XbaI |
| *NRAMP1-YR* | CCGCTCGAG GTCGATATTTTCGGTAGACC XholI |
| *MdBI-1-F* | CCGAGAAGAATGAGAAGAAG |
| *MdBI-1-R* | GGCAACACTAGACAATCAC |
| *MdBAG*-F | GTTGGTGTGATGCTTCTG |
| *MdBAG*-R | CCTGTAGTCTTCGTCTTCA |
| MdDAD-F | TGTCGGGTCATTCCCATTCA |
| MdDAD-R | CCCGTTCCGGTGCTAAATCC |
| MdVPEγ-F | CTCCCGAAGGATCACCAAGG |
| MdVPEγ-R | GCAGTCCCAGTCATCAACGA |
| MdACTIN-F | ACACGGGGAGGTAGTGACAA |
| MdACTIN-R | CCTCCAATGGATCCTCGTTA |

BamHI, KpnI, XbaI and XhoI the recognition sequences are underlined
